# Dyspnea Reduction With L‐Menthol Inhalation Improves High‐Intensity Endurance Running Performance in Healthy Trained Adults

**DOI:** 10.1002/ejsc.70262

**Published:** 2026-10-23

**Authors:** Thiago Ribeiro Lopes, Adriano de Almeida Pereira, Felipe Silva Gomes, Daniel Gonçalves Duarte, Rita Dulcivana Soares, Caroline Beato Soares, Giovana Beato Soares, Samuele Maria Marcora, Bruno Moreira Silva

**Affiliations:** ^1^ Laboratory of Exercise Physiology at the Olympic Center of Training and Research Department of Physiology Paulista School of Medicine Federal University of São Paulo São Paulo São Paulo Brazil; ^2^ São Paulo Association for Medicine Development São Paulo São Paulo Brazil; ^3^ Post‐Graduate Program in Translational Medicine Department of Medicine Paulista School of Medicine Federal University of São Paulo São Paulo São Paulo Brazil; ^4^ Post‐graduate Program in Pharmaceutical Sciences Federal University of Alfenas Alfenas Minas Gerais Brazil; ^5^ Faculty of Medicine Pontifical Catholic University of Minas Gerais Poços de Caldas Minas Gerais Brazil; ^6^ Department of Life Quality Studies University of Bologna Bologna Italy; ^7^ National Institute of Science and Technology—INCT Physical (In)activity and Health CNPq Brasília Brazil

**Keywords:** aerobic performance, breathlessness, exercise tolerance, middle‐distance running, psychobiology

## Abstract

Dyspnea occurs during high‐intensity running in healthy trained individuals and may limit endurance performance. Given that L‐menthol inhalation can alleviate dyspnea in other contexts, we investigated whether L‐menthol inhalation reduces dyspnea and improves endurance performance during high‐intensity running in healthy trained individuals. Nine females (mean ± standard deviation; $\dot{V}$O_2peak_ = 55.8 ± 6.8 mL/kg/min) and nine males ($\dot{V}$O_2peak_ = 60.3 ± 4.6 mL/kg/min) endurance runners with normal spirometry completed a randomized crossover counterbalanced trial with L‐menthol inhalation or a strawberry scent (placebo). Performance was measured with a treadmill time‐to‐exhaustion (TTE) test at 85% of the peak velocity achieved during a preliminary cardiopulmonary exercise test. Cardiorespiratory, metabolic, and perceptual responses were measured throughout. Compared with the placebo, L‐menthol inhalation reduced dyspnea across the TTE test (∼1 AU; *p* = 0.004) and extended the TTE test duration (406 ± 94 vs. 445 ± 106 s; *p* = 0.009). Pulmonary ventilation (*p* = 0.377), tidal volume (*p* = 0.298), O_2_ consumption (*p* = 0.277), CO_2_ production (*p* = 0.786), pulse O_2_ saturation (*p* = 0.766), and heart rate (*p* = 0.974) did not differ between conditions. Breathing frequency was lower with L‐menthol inhalation than with placebo (*p* = 0.045). L‐menthol inhalation attenuated dyspnea and enhanced high‐intensity running endurance performance without substantial changes in cardiorespiratory and metabolic responses to exercise. These results suggest that dyspnea constitutes an important constraint on endurance performance during high‐intensity running in healthy trained individuals, and identify acute L‐menthol inhalation as an effective ergogenic strategy for this exercise modality and population.

**Trial Registration:**

Registered at the Brazilian Registry of Clinical Trials (U1111‐1289‐3438)

## Highlights

- Inhaling L‐menthol during high‐intensity running reduced the breathing discomfort and allowed trained runners to sustain exercise for longer.
- These benefits occurred without meaningful changes in cardiorespiratory and metabolic responses to exercise, suggesting that breathing discomfort plays an important role as a limiting factor of endurance performance.
- L‐menthol inhalation may represent a simple and practical strategy to improve high‐intensity running endurance performance by alleviating breathing discomfort in trained individuals.

## 1 Introduction

High‐intensity exercise can place a substantial demand on the respiratory system of healthy trained individuals, resulting in pronounced levels of dyspnea, defined as breathing discomfort or unpleasantness (Ferguson et al. 2025; Muscat et al. 2015). This exertional dyspnea is frequently reported as a reason for exercise termination in trained individuals, and therefore presumably plays a significant role in limiting endurance performance (Killian, Leblanc, et al. 1992; Killian, Summers, et al. 1992; Muscat et al. 2015). However, previous studies were unable to experimentally test the isolated contribution of dyspnea to endurance performance, as reducing dyspnea simultaneously induced other physiological changes that could also influence performance outcomes (Harms et al. 1997; Powers et al. 1986; Ulrich et al. 2017; Verges et al. 2008). For example, respiratory muscle unloading, hyperoxia, or respiratory muscle training elicits improvement in blood flow and oxygen delivery to locomotor muscles, reducing muscle fatigue (Harms et al. 1997; Powers et al. 1986; Ulrich et al. 2017; Verges et al. 2008). Therefore, manipulations that relieve only dyspnea during high‐intensity exercise could reveal the isolated contribution of dyspnea to endurance performance and have practical relevance to training and competition.

Studies have shown that L‐menthol inhalation attenuated dyspnea during respiratory loading tasks (Kanezaki and Ebihara 2017; Kanezaki et al. 2020; Nishino et al. 1997). The underlying mechanism possibly involves the transient receptor potential melastatin 8 (TRPM8), responsible for sensing cold temperatures in the upper airways (Kanezaki et al. 2021). When activated by L‐menthol inhalation, this receptor appears to generate a higher perception of airflow during breathing, even without changes in the diameter of the upper airways or pulmonary function (Eccles et al. 1988; Eccles et al. 1987; Kanezaki and Ebihara 2017; Kanezaki et al. 2020). As the prevailing dyspnea model posits that exertional dyspnea arises from a mismatch between central ventilatory drive and actual ventilation (Sheel et al. 2011), such an increased airflow perception may change the central interpretation toward better matching, contributing to the observed alleviation of dyspnea during respiratory loading tasks (Kanezaki and Ebihara 2017; Kanezaki et al. 2020, 2021). Thus, inhaling L‐menthol may increase the perception of airflow and reduce dyspnea during high‐intensity exercise in healthy trained runners.

Previous studies have shown that L‐menthol inhalation can reduce dyspnea during moderate‐intensity cycling in healthy young adults and high‐intensity cycling in patients with chronic obstructive pulmonary disease (COPD), without altering cardiorespiratory and metabolic responses to exercise (Kanezaki and Ebihara 2017; Schaeffer et al. 2025). However, the effect of L‐menthol inhalation on endurance performance has not been investigated in healthy trained individuals. In patients with COPD, L‐menthol inhalation reduced dyspnea but did not improve endurance performance during high‐intensity cycling (Schaeffer et al. 2025). This result may relate to the fact that, during high‐intensity cycling, a primary reason for exercise cessation is perceived leg effort, even in patients with COPD (Killian, Leblanc, et al. 1992; Killian, Summers, et al. 1992; Murray et al. 2009). Conversely, during high‐intensity running, dyspnea appears to play a more relevant role in the decision to stop the exercise (Murray et al. 2009; Muscat et al. 2015), particularly in healthy trained individuals (Muscat et al. 2015).

Given this background, it remains to be determined whether L‐menthol inhalation can reduce dyspnea during high‐intensity running in healthy trained individuals, thereby improving endurance performance. To address this gap, the present study sought to examine cardiorespiratory, metabolic, perceptual, and performance responses to high‐intensity running under L‐menthol inhalation in trained healthy individuals. We hypothesized that, compared to placebo, L‐menthol inhalation would attenuate dyspnea during high‐intensity running and enhance endurance performance without altering cardiorespiratory or metabolic responses to exercise.

## 2 Materials and Methods

### 2.1 Subjects

Twenty‐one endurance runners volunteered to participate in the study. However, time‐to‐exhaustion data from three participants were identified as outliers and excluded (more details in the statistical analysis section). Thus, data from 18 participants were included in the final statistical analyses. We recruited subjects by disseminating information about the study to running teams in the city of São Paulo and through various social media platforms. The inclusion criteria involved being between 18 and 35 years old, training regularly (≥ 3 times/week, ≥ 6 months), with a recent 10‐km race time below 40 min for men and 45 min for women, not presenting with diagnosed diseases, and not using any chronic medication or supplementation. All participants were informed about the risks and discomforts associated with the experimental procedures and provided written informed consent before participating in the research. The study conformed to the Declaration of Helsinki and was approved by the Ethics Committee.

### 2.2 Experimental Design

Participants visited the laboratory on three occasions (Figure 1). During the first visit, we collected demographic information, anthropometric data, and training details. Thereafter, participants underwent pulmonary function and cardiopulmonary exercise testing (CPET; Supporting Information S1). Finally, participants were instructed and familiarized with all procedures and measurements that would be conducted in the subsequent experimental visits. During the second and third visits, participants performed an exercise protocol under two experimental conditions: inhaling L‐menthol (i.e., active intervention) or a strawberry scent (i.e., alternative placebo intervention). The order in which these interventions were administered to the participants was randomized (drawing lots) in a crossover, counterbalanced manner. Because participants could not be blinded to the intervention, we minimized the potential placebo effect on perceptual responses and endurance performance by withholding study hypotheses until the final visit. We also asked participants not to share their hypotheses with other team members. To avoid a possible experimenter effect, the researcher who provided verbal encouragement was blinded to the intervention administered during each experimental visit. The participants were instructed to reduce training load during the experiment period and refrain from exercising and consuming caffeine or alcohol for 24 hours before each visit. Given that the menstrual cycle phase does not appear to influence endurance performance (McNulty et al. 2020), we did not schedule the experimental visits according to a specific phase. Importantly, each female participant completed the experimental visits within the same menstrual cycle phase. The interval between visits was at least 24 hours and at most 7 days. The visits were conducted at the same time of day, with the ambient temperature set at 22°C. The study protocol was registered.

**FIGURE 1:**
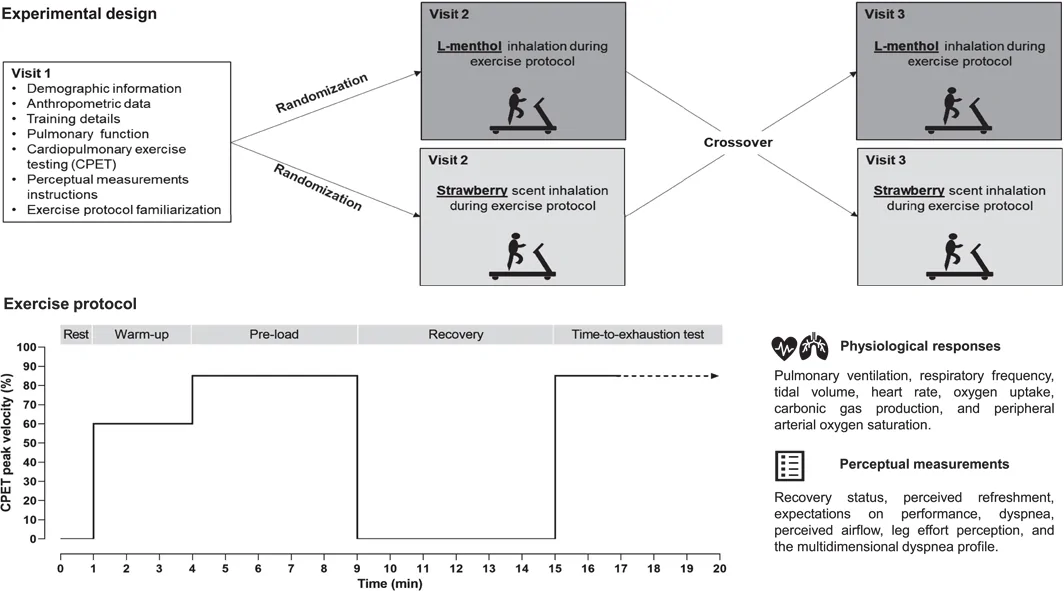
Study overview. Participants completed three visits. Visit 1 included participants' characterization assessments and familiarization with all procedures. Visits 2 and 3 involved an exercise protocol under two randomized (drawing lots), counterbalanced, and crossover experimental conditions: L‐menthol or strawberry scent (placebo) inhalation. The researcher delivering verbal encouragement was blinded to the intervention, and participants remained unaware of the study hypothesis until the end of the final visit. The interval between visits was at least 24 hours and at most 7 days. The Total Quality Recovery scale (perceived recovery status) was assessed upon laboratory arrival, whereas intervention expectations and baseline perceived refreshment were reported during the 1‐min rest phase at the beginning of the exercise protocol. Pulmonary ventilation, respiratory frequency, tidal volume, heart rate, oxygen uptake, and carbon dioxide production were continuously monitored and averaged every minute. During the pre‐load and time‐to‐exhaustion (TTE) tests, dyspnea, perceived airflow, and leg effort perception were recorded during the last 20 s of each minute and immediately at exhaustion. Following the pre‐load, the multidimensional dyspnea profile questionnaire was applied, while final perceived refreshment was rated again post‐exercise. Finally, peripheral arterial saturation was recorded 10 s before the rest period ended, and 1 min after both the pre‐load and TTE tests.

### 2.3 Interventions Preparation and Administration

Two ointments were prepared using petrolatum as a vehicle (Farmácia Herbanário Ltda., Brazil): one containing L‐menthol (300 mg/mL) and another containing strawberry scent (10 mg/mL). For the L‐menthol ointment, L‐menthol crystals (Batch number: MKCJ8767, Sigma‐Aldrich, Brazil) were triturated in a mortar with a pestle into a fine powder before being gradually incorporated into petrolatum under continuous mixing. The placebo ointment was prepared by directly incorporating the liquid strawberry essence (Batch number: 23^a^25‐B029‐100731, SM Empreendimentos Farmacêuticos Ltda., Brazil) into petrolatum using a mortar and pestle. All preparation procedures were performed under conditions that minimized the risk of cross‐contamination and microbial growth. The ointments were packaged in graduated syringes to ensure accurate dispensing of the required volume. For administration, 1 mL of the ointment was applied to the lower portion of an oro‐nasal silicone mask (V2 mask 7450 series, Hans Rudolph, USA), near the chin. This mask was subsequently used to measure respiratory and metabolic responses during the exercise protocol. Previous studies investigating the effect of L‐menthol inhalation on dyspnea adopted a comparable methodological approach (Kanezaki and Ebihara 2017; Nishino et al. 1997; Schaeffer et al. 2025).

### 2.4 Exercise Protocol

Participants underwent an exercise protocol that consisted of a 1‐min rest period, a 3‐min warm‐up at 60% of peak velocity (PV) from CPET, a 5‐min constant‐velocity phase at 85% of PV (pre‐load), a 5‐min recovery, and a subsequent time‐to‐exhaustion (TTE) test performed at 85% of PV (Figure 1). Treadmill grade was maintained at 1% (Jones and Doust 1996). The pre‐load was used to examine the effect of L‐menthol inhalation on dyspnea in a multidimensional manner, while controlling for both exercise intensity and duration. Next, the TTE test was used to assess endurance performance. In the TTE test, participants were instructed to sustain the exercise for as long as possible. Time was recorded with a manual stopwatch. Time recording was terminated upon volitional exhaustion, defined as the moment the participant requested to stop the test; as per prior instructions, this was signaled by grasping the front safety bar. Participants were verbally encouraged throughout the test by a researcher blinded to the experimental conditions. The same researchers conducted the exercise protocol for a given participant to minimize the effect that different people in the experimental room could have on participants' perceptual responses (Robertson and Noble 1997). Using an exercise protocol comparable to the present study, Laursen et al. 2007 reported a standard deviation of 60 s for the difference in time between two TTE tests performed 2–5 days apart. Therefore, the typical measurement error for this TTE is 42 s (Hopkins 2000).

### 2.5 Cardiorespiratory, Metabolic, and Perceptual Measures

The following respiratory and metabolic variables were obtained breath‐by‐breath (Quark CPET, Cosmed, Italy) throughout the exercise protocol: $\dot{V}$_E_, respiratory frequency (*f*_*R*_), tidal volume (*V*_*T*_), $\dot{V}$O_2_, and carbon dioxide production ($\dot{V}$CO_2_). HR was recorded beat‐by‐beat using a chest strap (H9, Polar, Finland). Data were averaged over 1‐min intervals using a customized Excel spreadsheet (Excel 2013, Microsoft, USA). To address incomplete final intervals in the TTE test, peak variables were averaged retroactively from the last 60 seconds of exercise. Arterial oxygen saturation was measured using a fingertip pulse oximeter (SpO_2_; Prince‐100C, Heal Force, Hong Kong). The device was placed on a finger during rest and was repositioned immediately after the pre‐load and the TTE test. SpO_2_ was recorded 10 s before finishing the rest period and 1 min after both the pre‐load and the TTE test.

Upon arrival, participants' recovery status was assessed using the 6 to 20 Total Quality Recovery (TQR) scale (Kenttä and Hassmén 1998). Expectations regarding the potential effects of the experimental interventions on TTE performance (positive, neutral, or negative) were reported during the 1‐min rest period at the beginning of the exercise protocol, during which participants were inhaling either L‐menthol or the strawberry scent. Perceived refreshment was rated during this period and after the exercise protocol. Dyspnea, perceived airflow, and leg effort perception were measured during the last 20 s of each exercise minute and immediately after exhaustion using a Portuguese‐validated Borg CR10 scale (Borg 1982), distributed via the Gunnar Borg platform (https://borgperception.se). Standardized instructions on the perceptual constructs and the scale use were provided before the exercise protocol (Ferguson et al. 2025; Lopes et al. 2022). During the recovery period after the pre‐load, dyspnea was assessed via the Portuguese validated version of the Multidimensional Dyspnea Profile (MDP) questionnaire (Belo et al. 2019). Participants were instructed to consider only the last minute of the previous exercise when answering the MDP questions. Details on perceptual measure assessment are in the Supporting Information S1.

### 2.6 Statistical Analysis

Based on a priori sample size calculation (Supporting Information S1), 15 participants were required to detect meaningful differences in dyspnea during the TTE test with L‐menthol inhalation compared to placebo, without taking into account a between‐sex comparison. However, considering the possibility of sample loss due to participant withdrawal, injury, or technical problems with data collection, we decided to recruit 21 volunteers for the present study. Cardiorespiratory, metabolic, and perceptual data from the pre‐load phase were analyzed using the 1‐min averages, whereas the TTE tests were analyzed at individual isotime points (25%, 50%, 75%, and 100%), according to Nicolò et al. 2019. For each participant, the final full minute of the shorter TTE test was defined as 100% individual isotime. Then, this time was multiplied by 0.25, 0.50, and 0.75, and when necessary, rounded to the nearest minute to determine the time corresponding to 25%, 50%, and 75% individual isotimes. Outliers were identified using two methods: the interquartile range (IQR) and the Z‐score (Hoaglin et al. 1986). The IQR method involved calculating the lower (Q1) and upper (Q3) quartiles, as well as the IQR (Q3 minus Q1), of absolute changes in the TTE test duration between conditions (i.e., L‐menthol minus placebo). Outliers were considered as values of absolute change that were lower or higher than 1.5 times the IQR. In the Z‐score method, outliers were considered absolute changes that were either lower or higher than three Z‐scores, taking into account the previously presented data on the mean and standard deviation of the difference in TTE between two TTE tests (reproducibility data) using a protocol (intensity and duration) and a sample comparable to those of the present investigation (Laursen et al. 2007).

Data were analyzed using generalized linear mixed models (GLMM) to account for both fixed and random effects in the data structure. We explored a range of model specifications to identify the best‐fitting model for each dependent variable. Model families and link functions were chosen based on the distributional properties of the outcome variables. For continuous outcomes, we compared models assuming normal and gamma distributions with the identity link function. For the expectation outcome, we specified a multinomial distribution with the logit link function. When supported by the data, random slope structures were tested to account for individual variability, including random effects for participants, time (or isotime), condition, and interaction. We also examined alternative residual covariance structures to model within‐subject correlation, including identity, diagonal, unstructured, AR(1), and ARMA(1,1) structures. Model selection was guided by the Akaike Information Criterion (AIC), with lower values indicating better model fit. Model assumptions and residual distributions were further evaluated through diagnostic tools, particularly quantile‐quantile (Q‐Q) plots. Final models were selected based on both statistical criteria and diagnostic evaluations to ensure appropriate fit and valid inference (Supporting Information S1: Tables S1–S6). Pairwise comparisons were performed using Holm's post hoc test. Between‐condition differences in sensory quality choice in the MDP questionnaire were assessed using McNemar's Exact Test. Statistical analyses were conducted on Jamovi (version 2.6.26.0, The Jamovi Project, Australia) and IBM SPSS Statistics (version 21, IBM Corp., USA). Results are presented as mean ± standard deviation (descriptive statistics) and estimated marginal means [95% confidence intervals] (inferential statistics). Statistical significance was set at *p* < 0.05.

## 3 Results

### 3.1 Outliers Identification

Both IQR and Z‐score analyses consistently identified the same three outliers (Supporting Information S1: Figure S1). These participants exhibited substantial discrepancies in TTE duration between trials that clearly exceeded the known reproducibility range. Such large discrepancies could not be plausibly attributed to any difference in recovery status or the effect of menthol inhalation. Therefore, data from these three outliers were removed from the final analyses, which comprised the remaining 18 participants (Table 1).

**TABLE 1:** Participants' characteristics.

	Females	Males	Total
Demographic			
Sample, *n*	9	9	18
Black, *n* (%)	1 (11)	5 (56)	6 (33)
Caucasian, *n* (%)	8 (89)	4 (44)	12 (67)
Age, years	25 ± 5	25 ± 7	25 ± 6
Anthropometric			
Height, cm	163.0 ± 3.3	179.0 ± 8.6	171.0 ± 10.5
Weight, kg	55.3 ± 4.2	70.7 ± 12.0	63.0 ± 11.8
Training			
Weekly training, number of sessions	6 ± 2	8 ± 3	7 ± 3
Distance/training session, km	9 ± 3	10 ± 4	9 ± 3
Weekly training distance, km	55 ± 16	63 ± 27	60 ± 23
Pulmonary function			
FVC, L	3.9 ± 0.5	5.4 ± 0.7	4.6 ± 1.0
FVC, % predicted	100 ± 12	100 ± 16	100 ± 13
FEV_1_, L	3.3 ± 0.4	4.6 ± 0.6	3.9 ± 0.8
FEV_1_, % predicted	102 ± 10	102 ± 17	102 ± 13
MVV, L/min	139.0 ± 13.5	187.0 ± 21.4	162.9 ± 30.2
MVV, % predicted	108 ± 11	97 ± 14	102 ± 13
CPET peak values			
$\dot{V}$O_2_, mL/kg/min	55.82 ± 6.80	60.27 ± 4.60	57.73 ± 6.18
$\dot{V}$O_2_, % predicted	138 ± 15	124 ± 8	132 ± 14
HR, bpm	181 ± 5	184 ± 14	183 ± 10
HR, % maximum age‐predicted	95 ± 3	97 ± 7	96 ± 5
Velocity, km/h	17.4 ± 1.2	20.7 ± 0.9	19.1 ± 2.0
$\dot{V}$_E_, L/min	102.9 ± 13.3	151.2 ± 20.8	127.0 ± 30.1
$\dot{V}$_E_, %MVV	76 ± 7	80 ± 11	78 ± 9
Dyspnea, AU	9.5 ± 1.3	9.9 ± 1.3	9.7 ± 1.3
Perceived leg effort, AU	9.6 ± 1.9	10.3 ± 0.9	10.0 ± 1.5

*Note:* Data are mean ± standard deviation. Predicted values calculated from the Brazilian population based on Neder et al. 1999, Pereira et al. 2007, Prata et al. 2018; Rossi Neto et al. 2020. Age‐predicted maximal HR calculated based on Tanaka et al. 2001.

Abbreviations: CPET, cardiopulmonary exercise test; FEV_1_, forced expiratory volume in one second; FVC, forced vital capacity; HR, heart rate; MVV, maximal voluntary ventilation estimated by (FEV_1_ x 37.5) + 15.8 (Neder et al. 1999); $\dot{V}$_E_, pulmonary ventilation; $\dot{V}$O_2_, oxygen consumption.

### 3.2 Recovery Status, Perceived Refreshment, and Intervention Expectation

Participants' recovery status did not differ between conditions (L‐menthol: 15 [14, 16] AU; placebo: 15 [14, 16] AU; *p* = 0.618). There was a significant effect of condition (*p* < 0.001) and condition × time interaction (*p* < 0.001), but not time (*p* = 0.118), for perceived refreshment. Post hoc comparisons showed higher values with L‐menthol inhalation than with placebo, both at rest (L‐menthol: 6.5 [5.1, 7.8] AU; placebo: 3.6 [2.5, 4.6] AU; *p* = 0.002) and after the TTE test (L‐menthol: 8.9 [6.9, 10.8] AU; placebo: 3.5 [2.3, 4.7] AU; *p* < 0.001). Additionally, perceived refreshment increased from rest to after the TTE test with L‐menthol inhalation (*p* = 0.009), but not with placebo (*p* = 0.945). L‐menthol inhalation generated more positive (L‐menthol: 66%; placebo: 33%; *p* = 0.003) and less neutral expectation (L‐menthol: 17%; placebo: 50%; *p* = 0.023) about endurance performance compared with placebo. No difference was observed between experimental conditions for the negative expectation (L‐menthol: 17%; placebo: 17%; *p* = 0.986).

### 3.3 Cardiorespiratory, Metabolic, and Perceptual Measures

Compared to placebo, L‐menthol inhalation significantly reduced dyspnea and *f*_*R*_ during the pre‐load but did not have a significant effect on perceived airflow, leg effort perception, $\dot{V}$_E_, *V*_*T*_, HR, $\dot{V}$O_2_, and $\dot{V}$CO_2_ (Table 2). No significant differences were observed between conditions in dyspnea unpleasantness, sensory quality (intensity and descriptor choice), or emotional responses (Table 3). Compared with the placebo, L‐menthol inhalation significantly reduced dyspnea, *f*_*R*_, and perceived airflow during the TTE test, but did not have a significant effect on perceived leg effort, $\dot{V}$_E_, *V*_*T*_, HR, $\dot{V}$O_2_, and $\dot{V}$CO_2_ (Figures 2 and 3). There was a significant effect of time (*p* = 0.007) on SpO_2_, but not of condition (*p* = 0.766) or condition × time interaction (*p* = 0.809). Post hoc comparisons for the main effect of time revealed that SpO_2_ decreased from rest (L‐menthol: 98 [97, 99] %; placebo: 98 [97, 99] %) to after the pre‐load (L‐menthol: 96 [94, 97] %; placebo: 96 [94, 98] %; *p* = 0.021) and after the TTE test (L‐menthol: 95 [93, 97] %; placebo: 95 [93, 97] %; *p* = 0.006). No differences were observed between the pre‐load and the TTE tests (*p* = 0.103).

**TABLE 2:** Perceptual, cardiorespiratory, and metabolic responses to the pre‐load phase.

	Dyspnea (AU)	Airflow (AU)	Leg effort (AU)	$\dot{V}$_E_ (L/min)	*f*_*R*_ (breaths/min)	*V*_*T*_ (L)	HR (bpm)	$\dot{V}$O_2_ (L/min)	$\dot{V}$CO_2_ (L/min)
1st minute									
L‐menthol	3.1 [2.3, 3.9]	2.8 [1.7, 3.9]	4.6 [3.6, 5.6]	79.6 [67.4, 91.8]	42 [38, 46]	2.0 [1.7, 2.3]	155 [150, 160]	2.9 [2.4, 3.3]	2.6 [2.2, 3.0]
Placebo	3.9 [2.9, 4.8]	3.0 [2.0, 3.9]	4.6 [3.7, 5.6]	80.1 [69.2, 91.1]	44 [39, 48]	1.9 [1.7, 2.2]	155 [149, 161]	2.9 [2.5, 3.4]	2.6 [2.2, 3.1]
2nd minute									
L‐menthol	3.4 [2.6, 4.2]	3.8 [2.7, 4.9]	5.1 [4.1, 6.1]	91.0 [78.7, 103.2]	44 [40, 49]	2.2 [1.9, 2.5]	162 [157, 167]	3.2 [2.7, 3.7]	3.1 [2.7, 3.6]
Placebo	4.1 [3.2, 5.0]	4.1 [3.1, 5.0]	5.3 [4.3, 6.2]	93.5 [82.5, 104.5]	47 [42, 51]	2.1 [1.8, 2.4]	164 [158, 170]	3.3 [2.9, 3.7]	3.2 [2.8, 3.7]
3rd minute									
L‐menthol	4.0 [3.1, 4.8]	4.6 [3.5, 5.7]	5.9 [4.9, 6.9]	98.4 [86.2, 110.7]	47 [43, 51]	2.2 [1.9, 2.6]	168 [163, 173]	3.4 [2.9, 3.8]	3.4 [3.0, 3.8]
Placebo	4.8 [3.8, 5.7]	5.1 [4.1, 6.0]	6.0 [5.1, 7.0]	99.5 [88.5, 110.6]	49 [45, 54]	2.2 [1.9, 2.5]	168 [161, 174]	3.4 [3.0, 3.8]	3.5 [3.0, 3.9]
4th minute									
L‐menthol	5.0 [4.1, 5.9]	5.2 [4.1, 6.3]	6.5 [5.4, 7.5]	103.0 [90.7, 115.2]	49 [45, 53]	2.2 [1.9, 2.6]	172 [167, 177]	3.4 [3.0, 3.9]	3.5 [3.1, 4.0]
Placebo	5.6 [4.6, 6.6]	5.7 [4.8, 6.7]	6.7 [5.7, 7.7]	104 [93.4, 115.5]	52 [47, 56]	2.2 [1.9, 2.5]	171 [165, 177]	3.5 [3.1, 3.9]	3.6 [3.2, 4.0]
5th minute									
L‐menthol	5.6 [4.7, 6.6]	6.2 [5.1, 7.3]	7.1 [6.1, 8.2]	105.6 [93.3, 117.9]	51 [47, 55]	2.2 [1.9, 2.6]	174 [170, 179]	3.5 [3.0, 3.9]	3.5 [3.1, 4.0]
Placebo	6.2 [5.2, 7.2]	6.5 [5.5, 7.5]	7.5 [6.5, 8.5]	108.2 [97.2, 119.3]	53 [49, 58]	2.2 [1.9, 2.5]	175 [168, 181]	3.5 [3.1, 4.0]	3.6 [3.2, 4.1]
*p*‐values									
Condition	0.008	0.182	0.476	0.234	0.003	0.159	0.937	0.336	0.333
Time	< 0.001	< 0.001	< 0.001	< 0.001	< 0.001	< 0.001	< 0.001	< 0.001	< 0.001
Interaction	0.983	0.775	0.940	0.749	0.797	0.910	0.881	0.918	0.967

*Note:* Data are estimated marginal means [95% confidence intervals]. Placebo refers to the inhalation of a strawberry scent. Data were analyzed by generalized mixed models (GLMM), followed by Holm's post hoc analysis when necessary.

Abbreviations: *f*_*R*_, respiratory frequency; HR, heart rate; $\dot{V}$_E_, pulmonary ventilation; $\dot{V}$CO_2_, carbon dioxide production; $\dot{V}$O_2_, oxygen consumption; *V*_*T*_, tidal volume.

**TABLE 3:** Multidimensional dyspnea profile.

	L‐menthol	Placebo	*p*‐values
Affective dimension			
Dyspnea unpleasantness, AU	7 [6, 7]	7 [6, 7]	0.881
Sensory quality intensity			
Respiratory work, AU	7 [6, 8]	7 [6, 8]	0.935
Air hunger, AU	7 [5, 8]	7 [5, 9]	0.340
Tightness, AU	5 [3, 6]	5 [3, 6]	0.289
Mental effort, AU	7 [6, 9]	7 [6, 9]	0.934
Breathing a lot, AU	6 [5, 8]	6 [5, 8]	0.614
Emotional responses			
Depressed, AU	2 [1, 3]	3 [2, 3]	0.111
Anxious, AU	5 [3, 6]	5 [4, 7]	0.115
Frustrated, AU	4 [2, 5]	4 [3, 5]	0.861
Angry, AU	3 [1, 4]	3 [1, 4]	0.911
Afraid, AU	2 [2, 3]	2 [2, 3]	0.695
Sensory quality choice			
Respiratory work, %	24	12	0.625
Air hunger,%	18	35	0.250
Tightness, %	6	12	1.000
Mental effort, %	29	29	1.000
Breathing a lot, %	24	12	0.625

*Note:* Data are estimated marginal means [95% confidence intervals]. Placebo refers to the inhalation of a strawberry fragrance. Generalized Mixed Models (GLMM) were used to analyze the affective dimension, sensory quality intensity, and emotional responses. The McNemar Exact Test was used to analyze sensory quality choice.

**FIGURE 2:**
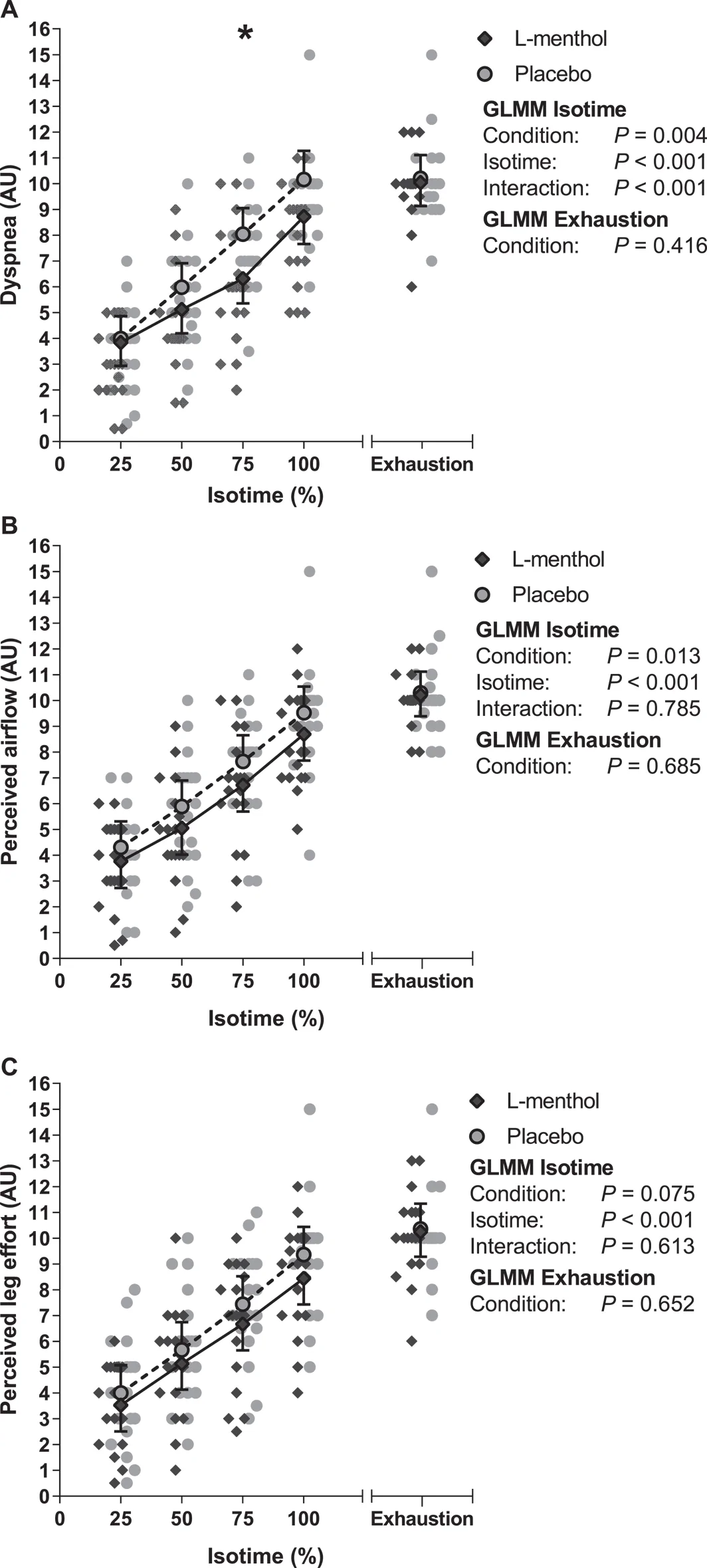
Effect of L‐menthol (active intervention) and strawberry scent (placebo intervention) inhalation on perceptual responses at individual isotime and exhaustion in the time‐to‐exhaustion (TTE) test. A hundred percent of individual isotime represents the last complete minute of the shorter TTE test for a given subject (L‐menthol or placebo). Panel A, perception of discomfort or unpleasantness of breathing (i.e., dyspnea). *, lower than placebo (*p* = 0.002). Panel B, perception of airflow through the airways. Panel C, perception of effort to command the legs during exercise. Data are presented as estimated marginal means (bordered symbols) and 95% confidence intervals (error bars). Individual participant responses are displayed as background symbols. Data were analyzed by generalized mixed models (GLMM), followed by Holm's post hoc analysis when necessary.

**FIGURE 3:**
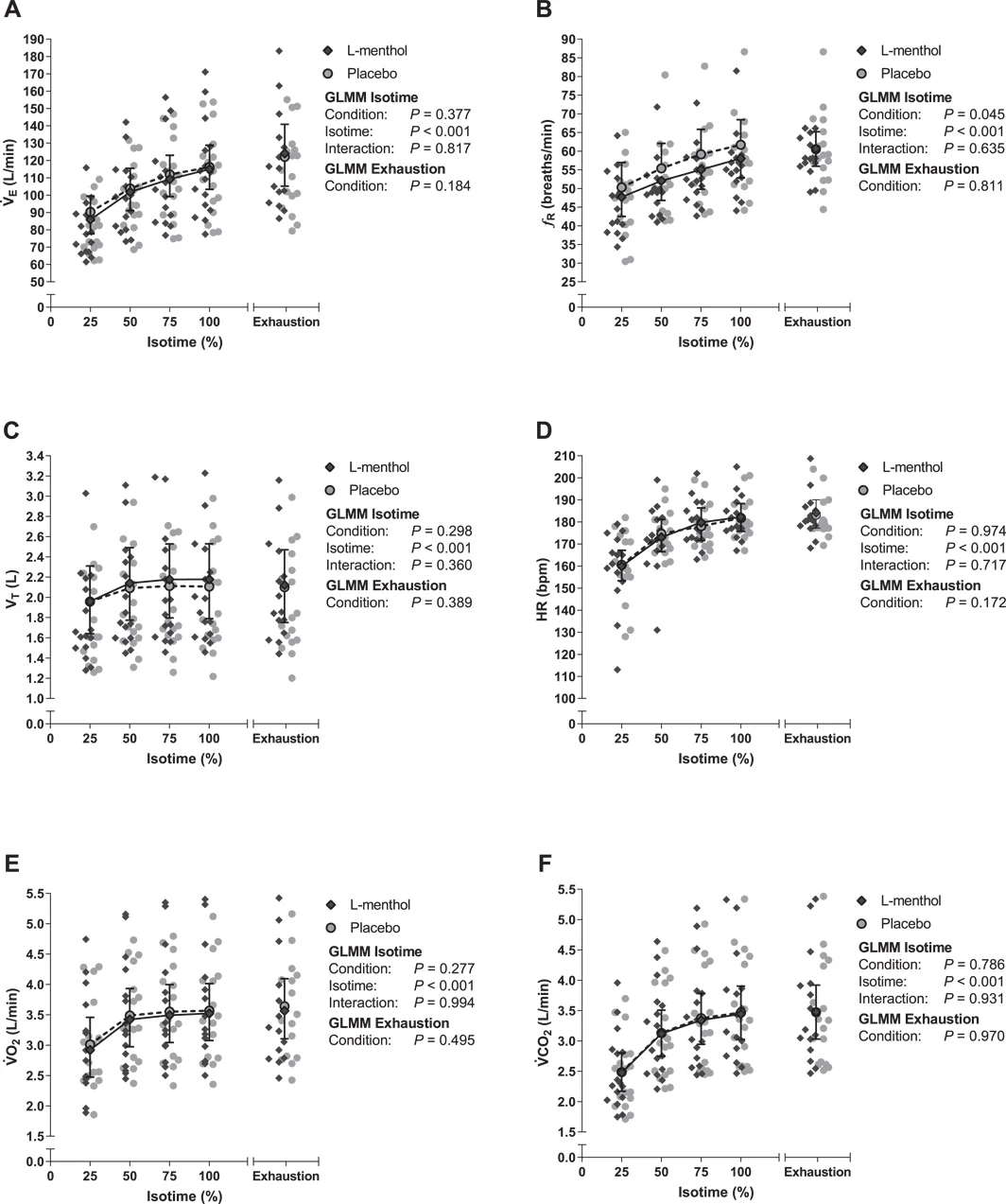
Effect of L‐menthol (active intervention) and strawberry scent (placebo intervention) inhalation on cardiorespiratory and metabolic responses at individual isotime and exhaustion in the time‐to‐exhaustion (TTE) test. A hundred percent of individual isotime represents the last complete minute of the shorter TTE test for a given participant (L‐menthol or placebo). Panel A, pulmonary ventilation. Panel B, respiratory frequency; panel C, tidal volume; panel D, heart rate; panel E, oxygen consumption. Panel F, carbon dioxide production. Data are presented as estimated marginal means (bordered symbols) and 95% confidence intervals (error bars). Individual participant responses are displayed as background symbols. Data were analyzed by generalized mixed models (GLMM), followed by Holm's post hoc analysis when necessary.

### 3.4 Endurance Performance

L‐menthol inhalation significantly increased TTE compared with placebo (mean difference = 39.4 [12.3, 66.4] s; *p* = 0.009; Figure 4). This effect remained significant after controlling for participants' expectations regarding the interventions (controlled mean difference = 32.9 [4.6, 61.1] s; *p* = 0.030). Nine of the 18 participants showed improvements in TTE that exceeded the typical measurement error.

**FIGURE 4:**
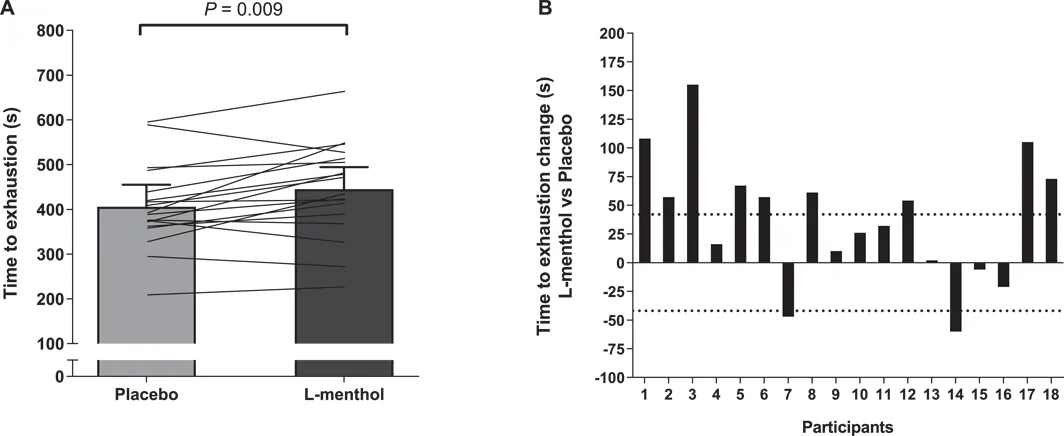
Effect of L‐menthol (active intervention) and strawberry scent (placebo intervention) inhalation on the time‐to‐exhaustion (TTE) test performance. Panel A, TTE test duration in each experimental condition. Data are presented as estimated marginal means (bars) and 95% confidence intervals (error bars). Paired lines illustrate individual participant responses across both conditions. Data were analyzed by generalized mixed models (GLMM). Panel B, absolute change in TTE test duration during L‐menthol inhalation compared to placebo in each participant. Dotted lines represent the typical measurement error (42 s) estimated from a previous study's data (Laursen et al. 2007), as suggested by Hopkins (Hopkins 2000).

## 4 Discussion

The principal findings of the present study were that L‐menthol inhalation reduced dyspnea during high‐intensity running and enhanced endurance performance in healthy trained individuals, without eliciting substantial changes in the cardiorespiratory and metabolic responses to exercise. Therefore, our results suggest that dyspnea plays an important role as a limiting factor of endurance performance during high‐intensity running in healthy trained individuals, and identify acute L‐menthol inhalation as an effective ergogenic strategy for this exercise modality and population.

### 4.1 Manipulation Checks

Our manipulation checks corroborate the validity of the experimental conditions. In the present study, participants began each condition with a similar level of perceived recovery status, suggesting proper management of training load and rest during the experiment period, which minimized any effect that incomplete recovery could have on outcomes. Moreover, participants perceived higher refreshment with L‐menthol inhalation than with the strawberry scent. Indeed, L‐menthol activates cold receptors (TRPM8) in afferent neurons from the trigeminal nerve in the upper airways (Kanezaki et al. 2021), causing a cooling sensation (Kanezaki and Ebihara 2017), which is perceived as refreshment (Eccles 2003). Importantly, perceived refreshment increased from rest to post‐exercise with L‐menthol inhalation, likely reflecting sustained activation of cold receptors in the upper airways during high‐intensity running. Despite using a strawberry scent as an alternative placebo intervention and blinding participants to the specific study objectives (Beedie et al. 2018), L‐menthol inhalation elicited more positive performance expectations than the strawberry scent. To address this (Beedie et al. 2018), we included performance expectations as covariates in the TTE analysis. This analysis revealed that, even when controlling for expectations, L‐menthol inhalation significantly improves endurance performance in healthy trained runners. This finding suggests that the ergogenic effect of L‐menthol inhalation is more than just a placebo effect.

### 4.2 Effect of L‐Menthol Inhalation on Dyspnea and Perceived Airflow

We observed a consistent dyspnea alleviation with L‐menthol inhalation during high‐intensity running. This relief sharpened near the TTE test completion, suggesting that L‐menthol's benefits scale with rising ventilatory demand. Our finding is consistent with the effect of L‐menthol inhalation on dyspnea found in other studies involving both healthy individuals and patients with COPD, using various dyspnea‐inducing tasks such as flow‐resistive and elastic‐loaded breathing, as well as moderate‐ and high‐intensity cycling (Kanezaki and Ebihara 2017; Kanezaki et al. 2020; Nishino et al. 1997; Schaeffer et al. 2025). L‐menthol inhalation is thought to alleviate dyspnea by “fooling” the brain, as it would enhance the perception of airflow, even without changing upper airway resistance or pulmonary function (Eccles 2003; Eccles et al. 1988; Eccles et al. 1987; Kanezaki et al. 2021). This hypothesis, however, was primarily based on studies that assessed perceived nasal airflow under L‐menthol inhalation at rest in the absence of dyspnea (Eccles et al. 1988; Eccles et al. 1987). To our knowledge, only two studies examined whether L‐menthol inhalation–induced dyspnea relief would be accompanied by an enhanced perception of airflow. Kanezaki et al. 2020 found a greater perceived airflow during a 2‐min inspiratory resistive task (facial mask) under L‐menthol inhalation. Schaeffer et al. 2025 observed that 85% of participants (patients with COPD) reported, after a high‐intensity cycling until exhaustion, that inspiratory airflow was easier during exercise with L‐menthol inhalation. Our results, in contrast, showed that perceived airflow during breathing remained unchanged during the pre‐load or even decreased during the TTE test under L‐menthol inhalation. These discrepancies may stem from the airflow sensation assessment, since perceptual measures are sensitive to the instructions given to assess them (Pageaux 2016), and there is no consensus on how to measure perceived airflow. While we asked participants to rate how much air passed through the airways during breathing, Kanezaki et al. 2020 requested participants to quantify the speed at which air passed during inspiration, and Schaeffer et al. 2025 assessed whether it was or was not easier to breathe in, rather than the perception of airflow itself.

Differences in the dyspnea‐inducing tasks may also contribute to the conflicting results observed for perceived airflow between the present and previous studies. It is plausible to assume that high‐intensity treadmill running likely generates a greater amount of sensorial information than breathing tasks and cycling exercise. For example, breathing tasks provide negligible lower‐limb sensory input, and stationary cycling, despite engaging the lower limbs, involves only modest balance‐related proprioceptive stimulation compared with treadmill running. The more complex sensory environment during high‐intensity treadmill running may prioritize processing exercise‐relevant sensations, potentially reducing the perceived airflow (Scalf et al. 2013). Therefore, our findings do not support the idea that a higher perception of airflow during breathing is responsible for relieving dyspnea during high‐intensity running under L‐menthol inhalation. Possible alternative explanations may be an alteration of the activity of brain regions involved in dyspnea genesis caused by trigeminal nerve stimulation with L‐menthol inhalation, a distraction effect caused by the cooling sensation, or a possible increased pleasure associated with the refreshment feeling (Aucoin et al. 2023; Eccles et al. 2013; Nishino et al. 1997). Future studies should investigate these alternative hypotheses.

We found that L‐menthol inhalation did not decrease dyspnea as measured by the MDP questionnaire after the pre‐load phase. However, during exercise, dyspnea measured using the CR10 scale was lower under L‐menthol inhalation. This difference may be related to the number of measurements affecting the variability of the results. The MDP questionnaire was administered only once after the pre‐load. In contrast, during the pre‐load, dyspnea was measured five times using the CR10 scale, which is not affected by potential recall bias and is the measurement of choice during exercise. In addition, no differences in sensory qualities or emotional responses in the MDP questionnaire were observed between L‐menthol inhalation and placebo in the present study. Schaeffer et al. 2025, in turn, found lower perceptions of air hunger and mental breathing effort but unchanged emotional responses under L‐menthol inhalation in patients with COPD. This difference may be related to the exercise test in which the MDP questionnaire was used. Schaeffer et al. 2025 applied it after exercise until exhaustion. Conversely, we assessed the MDP questionnaire responses after a fixed submaximal exercise duration to ensure that participants experienced a comparable exercise stimulus. However, by design, this stimulus had to be shorter than the effort required to reach exhaustion, which consequently elicited lower levels of dyspnea and may have obscured potential L‐menthol effects. Therefore, future studies should apply the MDP questionnaire under varied standardized exercise stimuli in healthy trained individuals to better elucidate the influence of L‐menthol on multiple dimensions of dyspnea.

### 4.3 Effect of L‐Menthol Inhalation on Cardiorespiratory and Metabolic Responses to Exercise and Endurance Performance

In the present study, $\dot{V}$_E_ and *V*_*T*_ did not differ between the experimental conditions. In contrast, *f*_*R*_ was lower during L‐menthol inhalation compared with the placebo condition. Previous studies have not observed changes in the ventilatory responses to cycling exercise with L‐menthol inhalation, either in healthy individuals or in patients with COPD (Kanezaki and Ebihara 2017; Schaeffer et al. 2025). However, our finding is consistent with evidence from studies in anesthetized animals under resting conditions showing that TRPM8 activation in the upper airways by L‐menthol inhalation can reduce *f*_*R*_ (Pallares et al. 2016; Plevkova et al. 2013). The alteration in breathing pattern in the present study is unlikely to have physiological relevance, given the modest magnitude of changes. Specifically, *f*_*R*_ decreased by ∼2 and ∼4 breaths/min during the pre‐load phase and TTE test, respectively. However, studies on the relationship between ratings of perceived exertion (RPE) and *f*_*R*_ during endurance exercise in healthy subjects suggest that such small changes in *f*_*R*_ may have perceptual relevance (Nicolò et al. 2016). Further studies are required to test the hypothesis that the reduction in *f*_*R*_ is causally linked to the reduction in dyspnea and the improvement in endurance performance induced by L‐menthol inhalation.

To our knowledge, the present study was the first to demonstrate that L‐menthol inhalation enhanced high‐intensity running endurance performance in healthy trained individuals. In patients with COPD, however, Schaeffer et al. 2025 did not observe improvement in high‐intensity cycling endurance performance under L‐menthol inhalation. These contrasting results may reflect the role of perceptual responses in limiting endurance performance between exercise modalities. During high‐intensity cycling, perceived leg effort plays a more relevant role than dyspnea as a reason to stop exercising, both in healthy individuals and patients with COPD (Killian, Leblanc, et al. 1992; Killian, Summers, et al. 1992; Murray et al. 2009). Thus, dyspnea reduction with inhaled L‐menthol in the study by Schaeffer et al. 2025 may not have been sufficient to improve endurance performance in some patients. Conversely, during high‐intensity running, dyspnea appears to contribute more significantly than perceived leg effort as a reason for exercise termination (Murray et al. 2009; Muscat et al. 2015), particularly in trained individuals (Muscat et al. 2015).

Experimental evidence suggests a possible role of dyspnea as a limiting factor on endurance performance. When dyspnea was reduced through respiratory muscle unloading, hyperoxia, or respiratory muscle training, an improvement in endurance performance was observed (Harms et al. 1997; Ulrich et al. 2017; Verges et al. 2008). However, these experimental strategies also induced physiological changes that could improve endurance performance independently of dyspnea relief—for example, improved blood flow and oxygen delivery to locomotor muscles, thereby reducing muscular fatigue (Harms et al. 1997; Powers et al. 1986; Ulrich et al. 2017; Verges et al. 2008). Importantly, such physiological alterations were also accompanied by changes in cardiorespiratory and metabolic responses to exercise (Harms et al. 1997; Powers et al. 1986; Ulrich et al. 2017; Verges et al. 2008). Since we observed, as in previous menthol studies (Kanezaki and Ebihara 2017; Schaeffer et al. 2025), no changes in $\dot{V}$_E_, HR, $\dot{V}$O_2_, $\dot{V}$CO_2_, or SpO_2_ with L‐menthol inhalation, our findings reinforce that the improvement in endurance performance by menthol inhalation likely stems primarily from dyspnea relief itself, confirming that dyspnea per se plays a relevant role as a limiting factor of endurance performance during high‐intensity running in healthy trained individuals. Notably, despite a group‐mean improvement (39.4 s) below the TTE test typical measurement error (42 s), half the sample (*n* = 9) exceeded this threshold, demonstrating a robust ergogenic response. Crucially, this average acute enhancement is comparable to caffeine effects on running TTE tests (Wang et al. 2023). Moreover, such gain sustained across training sessions could accumulate over time, driving greater adaptations (Wu and Jiang 2024). Future studies should investigate the factors underlying the inter‐individual variability in L‐menthol inhalation responsiveness and evaluate its potential to amplify long‐term training outcomes.

### 4.4 Limitations

We assessed multiple perceptual responses throughout the exercise protocol. To minimize response bias, we employed comprehensive anchoring procedures and standardized instructions. While repeated assessments using a fixed order could potentially introduce order effects, any resulting error would likely be systematic, equally affecting both the L‐menthol and placebo trials. Nevertheless, these concurrent assessments precluded measuring some psychological variables that could be relevant in the context of the current study. For instance, subjective thermal strain can independently limit endurance performance (Van Cutsem et al. 2019). However, the effect of inhaled L‐menthol on subjective thermal strain during exercise in a thermoneutral environment remains unknown. Moreover, two recent meta‐analyses have indicated that other forms of L‐menthol administration (e.g., ingestion, creams, or gels) generally do not enhance endurance performance, despite reducing subjective thermal strain during exercise (Gavel et al. 2024; Keringer et al. 2020). Thus, we believe that any potential improvement in the subjective thermal strain from L‐menthol inhalation is unlikely to contribute to the effects observed on endurance performance in the present investigation. Finally, as endurance performance is multifactorial, dyspnea's isolated role must be interpreted cautiously alongside other psychophysiological determinants. For example, perceived exertion and motivation have been considered the main psychological constructs limiting endurance performance (Marcora 2019). Perceived exertion represents how hard an exercise is (Lopes et al. 2022; Pageaux 2016). It depends on the effort sensations to drive the locomotor muscles and to breathe (Lopes et al. 2022; Pageaux 2016). We measured only the perceived leg effort during high‐intensity running, which tended to be lower during the TTE test under L‐menthol inhalation compared with the placebo. It is thus plausible that L‐menthol inhalation may also reduce perceived exertion during exercise. Moreover, despite lacking motivation assessments, consistent peak psychophysiological responses suggest comparable maximal volitional exhaustion across trials, minimizing potential bias. Therefore, future studies should investigate the relationship between L‐menthol inhalation, dyspnea, perceived exertion, motivation, and endurance performance.

## 5 Conclusion

L‐menthol inhalation reduced dyspnea during high‐intensity running in healthy trained individuals, enhancing endurance performance without substantial changes in the cardiorespiratory and metabolic responses to exercise. The findings indicate that dyspnea plays an important role in limiting high‐intensity running endurance performance in healthy trained individuals, and L‐menthol inhalation has emerged as an ergogenic strategy that may benefit endurance runners during training and competition.

## Author Contributions

Thiago Ribeiro Lopes, Adriano de Almeida Pereira, and Bruno Moreira Silva conceived and designed the study. Thiago Ribeiro Lopes, Adriano de Almeida Pereira, Felipe Silva Gomes, Daniel Gonçalves Duarte, Rita Dulcivana Soares, Caroline Beato Soares, Giovana Beato Soares, Samuele Maria Marcora, and Bruno Moreira Silva performed material preparation, data collection, and results interpretation. Thiago Ribeiro Lopes and Adriano de Almeida Pereira wrote the first draft of the manuscript. All authors commented on previous versions of the manuscript. All authors have read and approved the final manuscript.

## Funding

Funding for the replacement of a component of the Quark CPET system was provided by the São Paulo Research Foundation (FAPESP, process no. 2023/12845‐7). For open access purposes, the authors have assigned the Creative Commons CC BY license to any accepted version of the article.

## Ethics Statement

The study conformed to the Declaration of Helsinki and was approved by the Ethics Committee of the Federal University of São Paulo (*n*°. 0974/2022).

## Consent

All participants were informed about the risks and discomforts associated with the experimental procedures and provided written informed consent before participating in the research.

## Conflicts of Interest

Rita Dulcivana Soares is the owner of the pharmacy that prepared the ointments used in the experimental interventions (Farmácia Herbanário Ltda., Brazil).

## Supporting information


Supporting Information S1

## Data Availability

Data are available upon request from the corresponding author.
