# Supplementary material for: Dyspnea Reduction With L‐Menthol Inhalation Improves High‐Intensity Endurance Running Performance in Healthy Trained Adults
Source: Eur J Sport Sci. 2026 Oct 23;26(11):e70262. doi: 10.1002/ejsc.70262 (PMC13691925; doi:10.1002/ejsc.70262)
Supplement: Supplementary file 1 [file EJSC-26-e70262-s001.pdf]

Supporting Information

Pulmonary function

Spirometry was performed using a calibrated turbine pneumotachometer (Quark CPET, Cosmed, Italy) with participants seated and wearing a nasal clip. Before testing, the assessor explained the respiratory maneuver in detail. Each participant attempted three to five maneuvers. From the best trial, forced vital capacity (FVC) and forced expiratory volume in one second (FEV_1) were obtained. Maximal voluntary ventilation (MVV) was estimated using the formula: (FEV_1 × 37.5) + 15.8 (Neder et al., 1999). FVC, FEV_1, and MVV values were expressed in absolute units and as percentages of predicted values for the Brazilian population (Neder et al., 1999; Pereira et al., 2007; Prata et al., 2018).

Cardiopulmonary exercise testing

The participants performed the CPET on a treadmill (Master ATL, Inbramed, Brazil). The protocol began with a 1-minute resting period, followed by a 3-minute warm-up at 10 km/h for females and 12 km/h for males. Thereafter, velocity increased 1 km/h until voluntary exhaustion. A 3-minute cool-down at 5 km/h finalized the test. Treadmill grade was maintained at 1% throughout the test. Cardiorespiratory and metabolic variables were measured breath-by-breath (Quark CPET, Cosmed, Italy). These data were filtered and averaged every 20 s (Quark CPET suite 10.0e, Cosmed, Italy). Dyspnea and perceived leg effort were quantified with a Portuguese validated version of the Category-Ratio 10 (CR10) Borg scale (https://borgperception.se) at the final 10 s of each minute and immediately after exhaustion when the participant did not complete a full minute. Standardized instructions were explained to the participant before the test, in accordance with previous recommendations (Ferguson et al., 2025; Lopes et al., 2022). Peak values for oxygen uptake ($\dot{V}O_2$), pulmonary ventilation ($\dot{V}_E$), and heart rate (HR) were considered as the highest values among 20-second averages, whereas peak velocity (PV) was considered as the final velocity sustained for at least 1 min. $\dot{V}O_2$ peak values were expressed in absolute units and as percentages of predicted values for the Brazilian population (Rossi Neto et al., 2020). HR peak values were expressed in absolute units and as percentages of age-predicted maximal HR values (Tanaka et al., 2001).

Perceptual measures

Expectation was questioned as “what this scent can do to endurance performance: improve, have no effect, or worsen?”. Perceived refreshment was questioned as “how refreshing the scent feels?”. Perceived airflow was questioned as “how much air is passing through the airways during breathing?”. Dyspnea was questioned as “how uncomfortable or unpleasant it is to breathe?” (Ferguson et al., 2025). To differentiate breathing intensity from discomfort or unpleasantness, participants were instructed to use a radio analogy: volume reflected intensity, while music style reflected discomfort or unpleasantness (Ferguson et al., 2025). Leg effort perception was questioned as “how hard or laborious it is to drive the legs during running?” (Lopes et al., 2022). The MDP questionnaire assessed the unpleasantness level of dyspnea (Belo et al., 2019), including related sensory qualities (i.e., respiratory work, air hunger, chest tightness, mental effort, and breathing a lot) and emotional responses (i.e., depression, anxiety, frustration, anger, and fear).

Sample size calculation

The input parameters to estimate the sample size were the following: effect size = 0.91, t-test type = paired sample, tails = two-tailed, p-value = 0.05, and power = 0.90. The effect size of interest was calculated by performing a random-effects meta-analysis from previous studies that investigated the effect of L-menthol inhalation on dyspnea in healthy adults (Kanezaki & Ebihara, 2017; Kanezaki et al., 2020; Nishino et al., 1997). Type I and Type II error rates were selected to minimize the risk of false-positive findings while maintaining high sensitivity to detect the expected effect. The calculations were performed on Jamovi (version 2.6.26.0, The Jamovi Project, Australia) using the "jpower" and "major" modules.

References

Belo, L. F., Rodrigues, A., Vicentin, A. P., Paes, T., de Castro, L. A., Hernandes, N. A., & Pitta, F. (2019). A breath of fresh air: Validity and reliability of a Portuguese version of the Multidimensional Dyspnea Profile for patients with COPD. PLoS One, 14(4), e0215544.

Ferguson, O. N., Jensen, D., Guenette, J. A., & Lewthwaite, H. (2025). Multidimensional assessment of breathlessness during exercise: current methods and recommendations. Respir Physiol Neurobiol, 336, 104456.

Kanezaki, M., & Ebihara, S. (2017). Effect of the cooling sensation induced by olfactory stimulation by L-menthol on dyspnoea: a pilot study. Eur Respir J, 49(4), 1601823.

Kanezaki, M., Terada, K., & Ebihara, S. (2020). Effect of Olfactory Stimulation by L-Menthol on Laboratory-Induced Dyspnea in COPD. Chest, 157(6), 1455-1465.

Lopes, T. R., Pereira, H. M., & Silva, B. M. (2022). Perceived Exertion: Revisiting the History and Updating the Neurophysiology and the Practical Applications. Int J Environ Res Public Health, 19(21), 14439.

Neder, J. A., Andreoni, S., Lerario, M. C., & Nery, L. E. (1999). Reference values for lung function tests. II. Maximal respiratory pressures and voluntary ventilation. Braz J Med Biol Res, 32(6), 719-727.

Nishino, T., Tagaito, Y., & Sakurai, Y. (1997). Nasal inhalation of l-menthol reduces respiratory discomfort associated with loaded breathing. Am J Respir Crit Care Med, 156(1), 309-313.

Pereira, C. A., Sato, T., & Rodrigues, S. C. (2007). New reference values for forced spirometry in white adults in Brazil. J Bras Pneumol, 33(4), 397-406.

Prata, T. A., Mancuzo, E., Pereira, C. A. C., Miranda, S. S., Sadigursky, L. V., Hirotsu, C., & Tufik, S. (2018). Spirometry reference values for Black adults in Brazil. J Bras Pneumol, 44(6), 449-455.

Rossi Neto, J. M., Tebexreni, A. S., Alves, A. N. F., Abreu, F. B., Nishio, P. A., Thomazi, M. C., . . . Smanio, P. E. P. (2020). VO2max-Based Physical Fitness Categories in a Brazilian Population with Supposed High Socioeconomic Status and without Structural Heart Disease. Arq Bras Cardiol, 115(3), 468-477.

Tanaka, H., Monahan, K. D., & Seals, D. R. (2001). Age-predicted maximal heart rate revisited. J Am Coll Cardiol, 37(1), 153-156.

Table S1. Final generalized linear mixed models structure for recovery status, perceived refreshment, intervention expectation, and arterial oxygen saturation.

Variable	Fixed Effects	Random Effects	Residuals Structure	Distribution	Link Function
Recovery status	Condition	Participant	Identity	Gamma	Identity
Perceived refreshment	Condition<br>Time<br>Interaction	Participant<br>Condition<br>Time	Identity	Gamma	Identity
Intervention expectation	Condition	Participant	Not applicable	Multinominal	Logit
Arterial oxygen saturation	Condition<br>Time<br>Interaction	Participant<br>Time	AR(1)	Gaussian	Identity

Note: For all models, the random effects covariance structure was kept at the software default (correlated).

Table S2. Final generalized linear mixed models structure for perceptual, cardiorespiratory, and metabolic responses during the pre-load phase.

**Variable**	**Fixed Effects**	**Random Effects**	**Residuals Structure**	**Distribution**	**Link Function**
Dyspnea	Condition<br>Time<br>Interaction	Participant<br>Condition	Identity	Gamma	Identity
Perceived airflow	Condition<br>Time<br>Interaction	Participant<br>Condition	AR(1)	Gaussian	Identity
Perceived leg effort	Condition<br>Time<br>Interaction	Participant<br>Condition	Identity	Gamma	Identity
Pulmonary ventilation	Condition<br>Time<br>Interaction	Participant<br>Condition	Identity	Gamma	Identity
Respiratory frequency	Condition<br>Time<br>Interaction	Participant<br>Condition	Identity	Gamma	Identity
Tidal volume	Condition<br>Time<br>Interaction	Participant<br>Condition	Identity	Gamma	Identity
Heart rate	Condition<br>Time<br>Interaction	Participant<br>Condition	AR(1)	Gaussian	Identity
Oxygen consumption	Condition<br>Time<br>Interaction	Participant<br>Condition	Identity	Gaussian	Identity
Carbon dioxide production	Condition<br>Time<br>Interaction	Participant<br>Condition	Identity	Gaussian	Identity

Note: For all models, the random effects covariance structure was kept at the software default (correlated).

Table S3. Final generalized linear mixed models structure for the Multidimensional Dyspnea Profile questionnaire.

**Variable**	**Fixed Effects**	**Random Effects**	**Residuals Structure**	**Distribution**	**Link Function**
Dyspnea unpleasantness	Condition	Participant	Identity	Gaussian	Identity
Respiratory work	Condition	Participant	Identity	Gamma	Identity
Air hunger	Condition	Participant	Identity	Gamma	Identity
Tightness	Condition	Participant	Identity	Gamma	Identity
Mental effort	Condition	Participant	Identity	Gamma	Identity
Breathing a lot	Condition	Participant	Identity	Gamma	Identity
Depressed	Condition	Participant	Identity	Gamma	Identity
Anxious	Condition	Participant	Identity	Gamma	Identity
Frustrated	Condition	Participant	Identity	Gamma	Identity
Angry	Condition	Participant	Identity	Gaussian	Identity
Afraid	Condition	Participant	Identity	Gamma	Identity

Note: For all models, the random effects covariance structure was kept at the software default (correlated).

Table S4. Final generalized linear mixed models structure for perceptual, cardiorespiratory, and metabolic responses over isotime during the time-to-exhaustion test.

Variable	Fixed Effects	Random Effects	Residuals Structure	Distribution	Link Function
Dyspnea	Condition<br>Isotime<br>Interaction	Participant<br>Condition	Identity	Gamma	Identity
Perceived airflow	Condition<br>Isotime<br>Interaction	Participant<br>Condition	AR(1)	Gaussian	Identity
Perceived leg effort	Condition<br>Isotime<br>Interaction	Participant<br>Condition	AR(1)	Gaussian	Identity
Pulmonary ventilation	Condition<br>Isotime<br>Interaction	Participant<br>Condition	Identity	Gamma	Identity
Respiratory frequency	Condition<br>Isotime<br>Interaction	Participant<br>Condition	Identity	Gamma	Identity
Tidal volume	Condition<br>Isotime<br>Interaction	Participant<br>Condition	Identity	Gamma	Identity
Heart rate	Condition<br>Isotime<br>Interaction	Participant	AR(1)	Gaussian	Identity
Oxygen consumption	Condition<br>Isotime<br>Interaction	Participant	Identity	Gaussian	Identity
Carbon dioxide production	Condition<br>Isotime<br>Interaction	Condition<br>Isotime<br>Interaction	Identity	Gaussian	Identity

Note: For all models, the random effects covariance structure was kept at the software default (correlated).

Table S5. Final generalized linear mixed models structure for perceptual, cardiorespiratory, and metabolic peak values in the time-to-exhaustion test.

**Variable**	**Fixed Effects**	**Random Effects**	**Residuals Structure**	**Distribution**	**Link Function**
Dyspnea	Condition	Participant	Identity	Gamma	Identity
Perceived airflow	Condition	Participant	Identity	Gamma	Identity
Perceived leg effort	Condition	Participant	Identity	Gamma	Identity
Pulmonary ventilation	Condition	Participant	Identity	Gamma	Identity
Respiratory frequency	Condition	Participant	Identity	Gaussian	Identity
Tidal volume	Condition	Participant	Identity	Gamma	Identity
Heart rate	Condition	Participant	Identity	Gamma	Identity
Oxygen consumption	Condition	Participant	Identity	Gaussian	Identity
Carbon dioxide production	Condition	Participant	Identity	Gaussian	Identity

Note: For all models, the random effects covariance structure was kept at the software default (correlated).

Table S6. Final generalized linear mixed models structure for the duration of the time-to-exhaustion test.

Variable	Fixed Effects	Random Effects	Residuals Structure	Distribution	Link Function
Duration	Condition	Participant	Identity	Gaussian	Identity
Expectation-adjusted duration	Condition	Participant	Identity	Gaussian	Identity

Note: In the expectation-adjusted duration model, participants' expectations regarding the interventions were entered as a covariate.

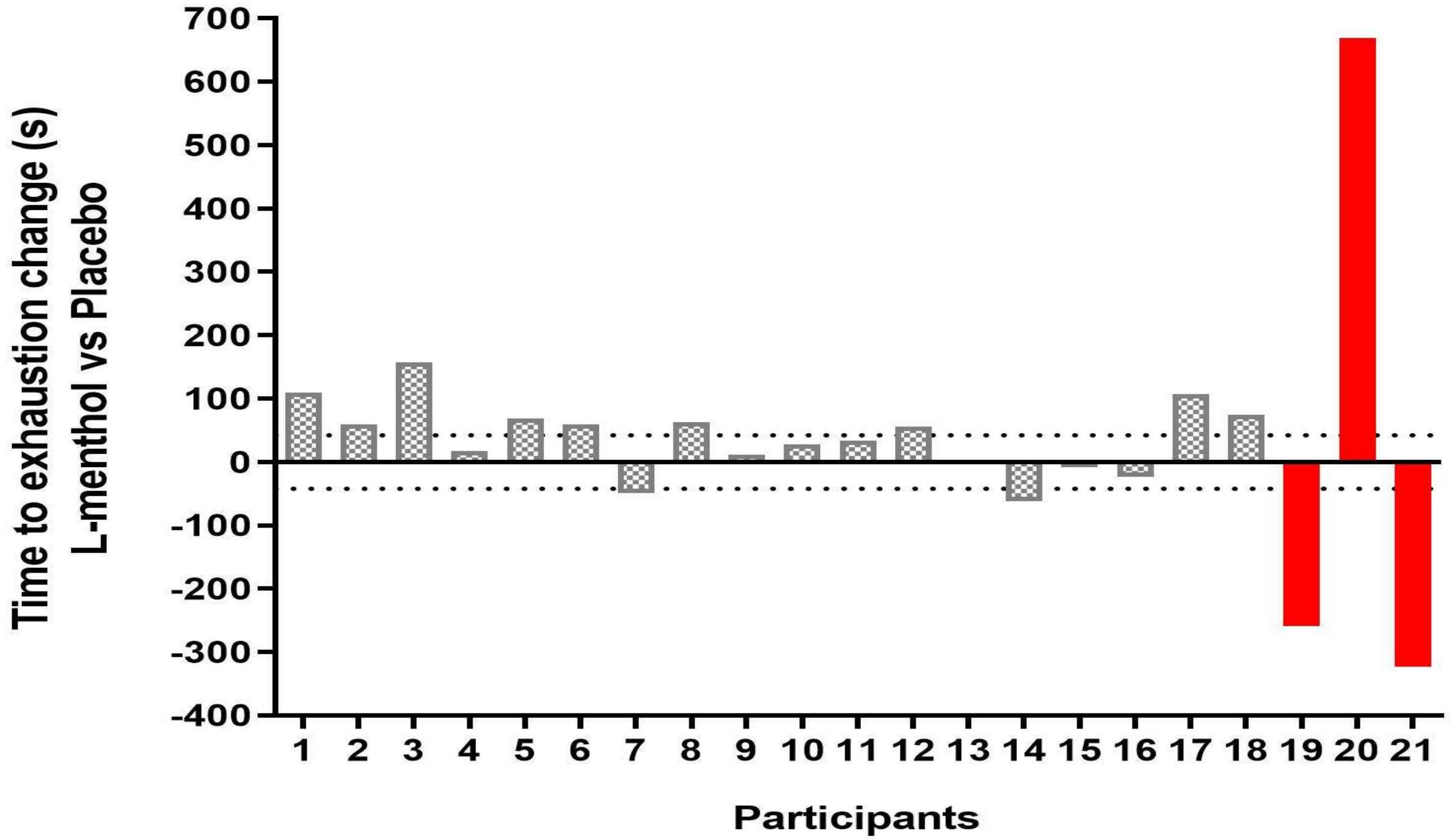

Figure S1. Absolute change in the time-to-exhaustion (TTE) test duration during L-menthol inhalation compared to placebo (strawberry scent) in each participant. Dotted lines represent the typical measurement error estimated from previous studies (see text for more details). Red bars represent outliers. Outliers were identified by two methods: the interquartile range (IQR) and the Z-score. The IQR method involved calculating the lower (Q1) and upper (Q3) quartiles, as well as the IQR (Q3 minus Q1), of absolute changes in the TTE test duration between conditions (i.e., L-menthol minus placebo). Outliers were considered as values of absolute change that were lower or higher than 1.5 times the IQR. In the Z-score method, outliers were considered absolute changes that were either lower or higher than three Z-scores, taking into account the previously presented data on the mean and standard deviation of the difference in TTE between two TTE tests (reproducibility data) using a protocol (intensity and duration) and a sample comparable to those of the present investigation (see text for more details).
